# Supplementary material for: A 28-Day Repeated Oral Administration Study of Mechanically Fibrillated Cellulose Nanofibers According to OECD TG407
Source: Nanomaterials (Basel). 2024 Jun 24;14(13):1082. doi: 10.3390/nano14131082 (PMC11242936; doi:10.3390/nano14131082)

**Table S1.** Individual body weights (g) of male rats at week 4. R indicates values measured during the recovery examination.


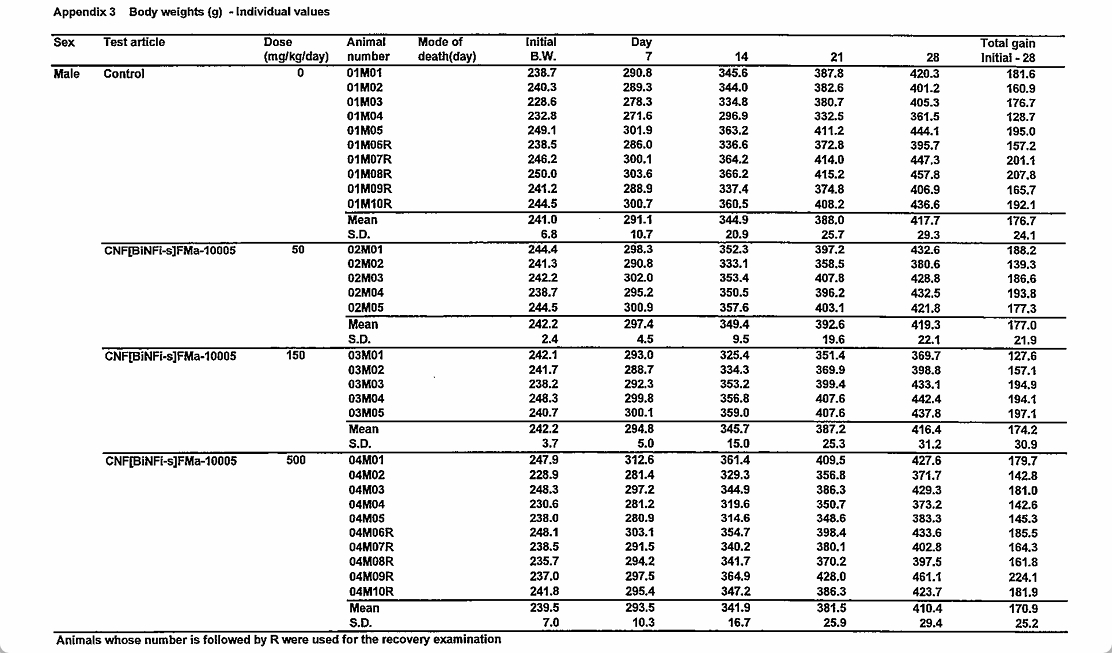


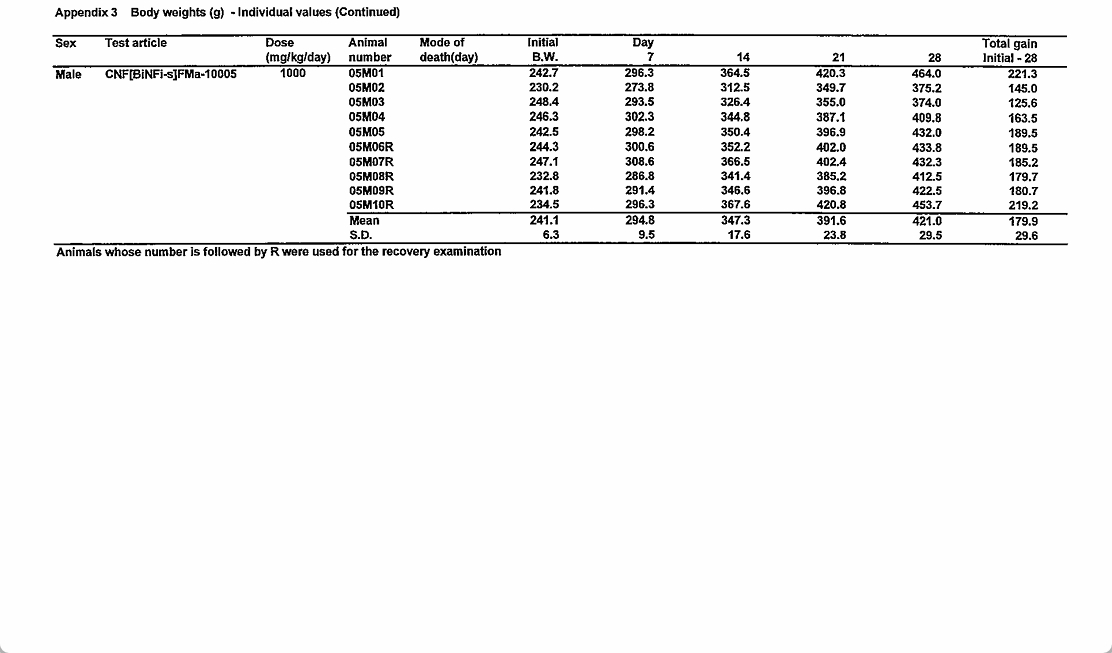


**Table S2.** Individual body weights (g) of male rats during the 2-week recovery period. R indicates values measured during the recovery examination.


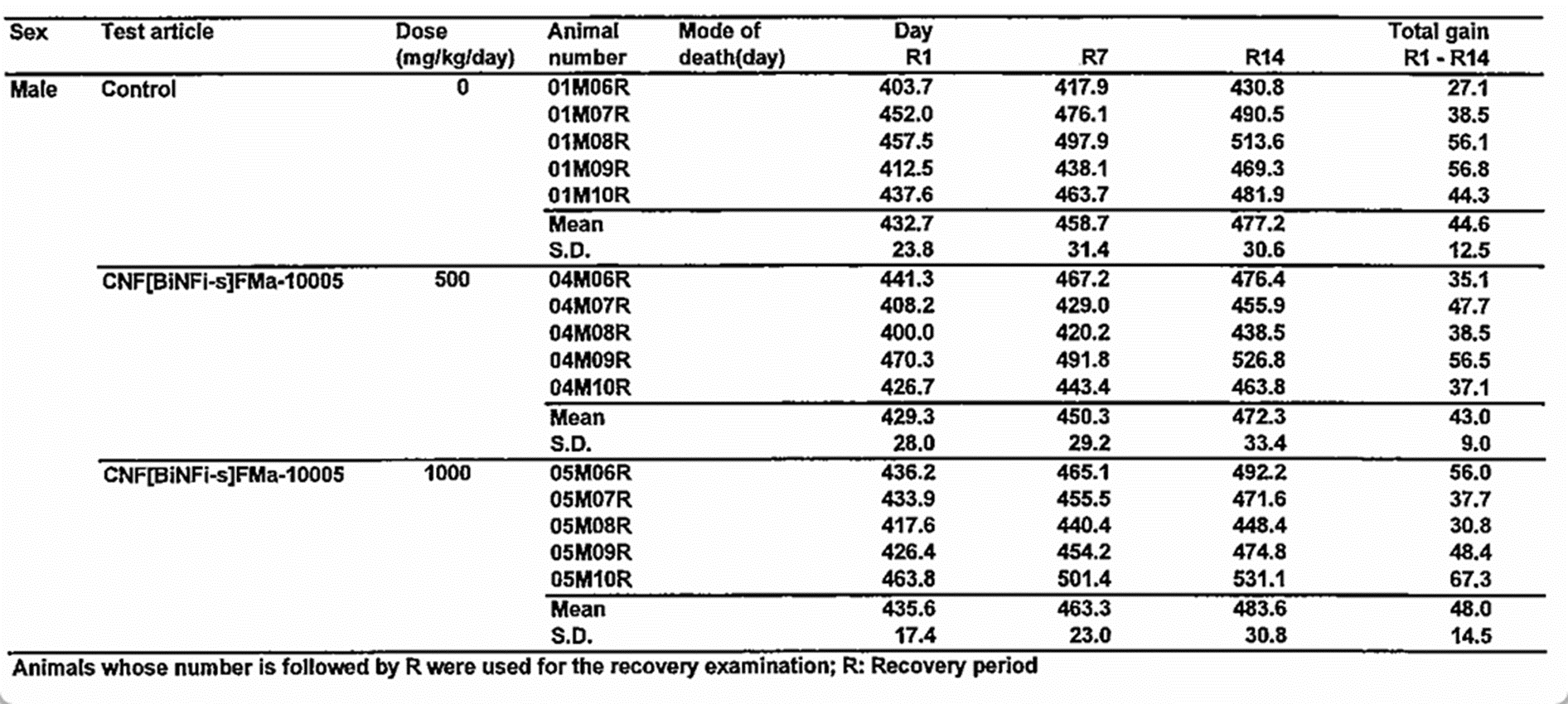


**Table S3.** Individual body weights (g) of female rats. R indicates values measured during the recovery examination.


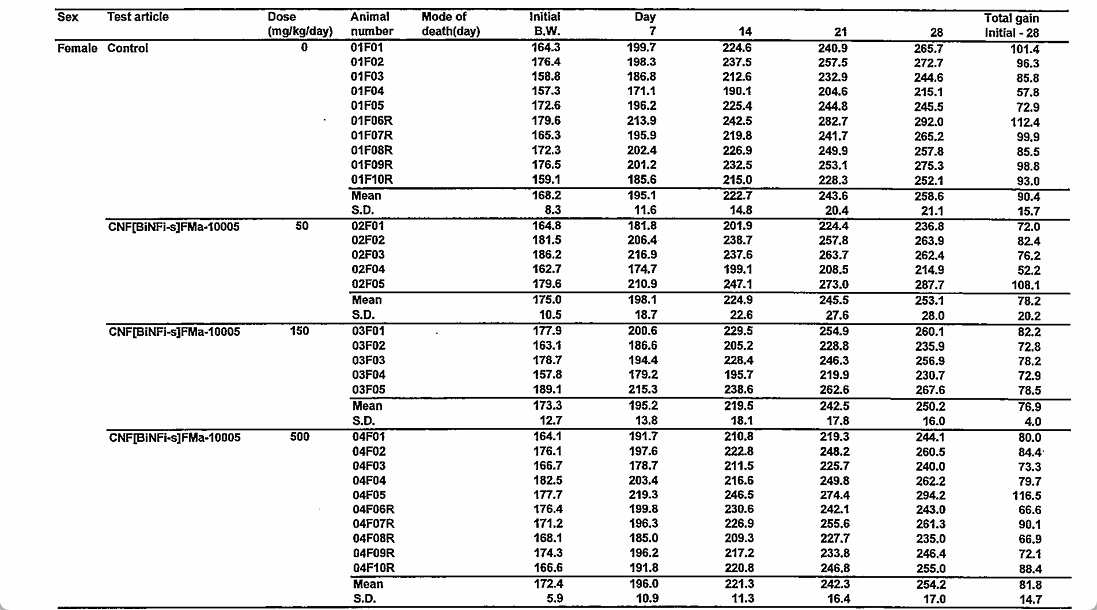


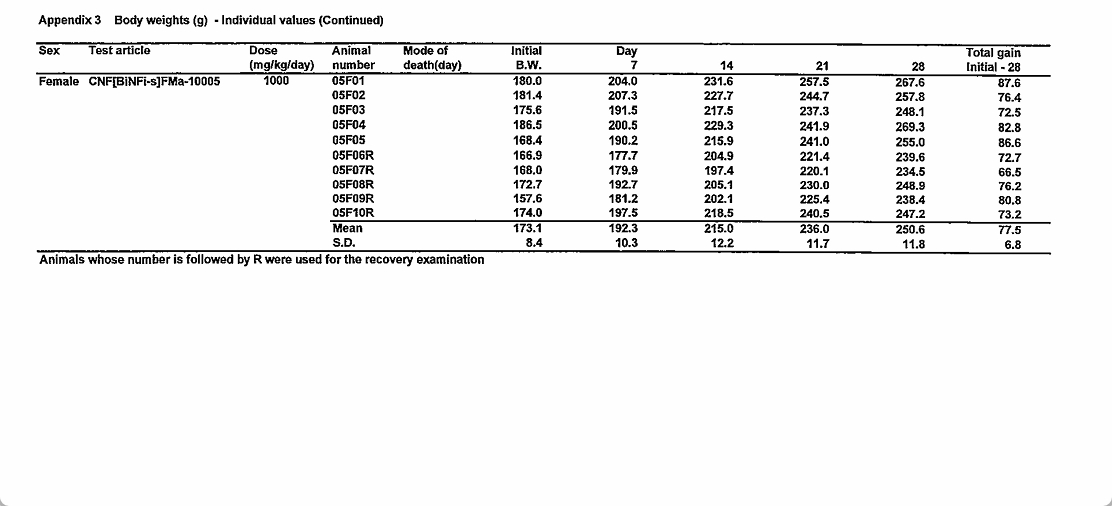


**Table S4.** Individual body weights (g) of female rats during the 2-week recovery period. R indicates values measured during the recovery examination.


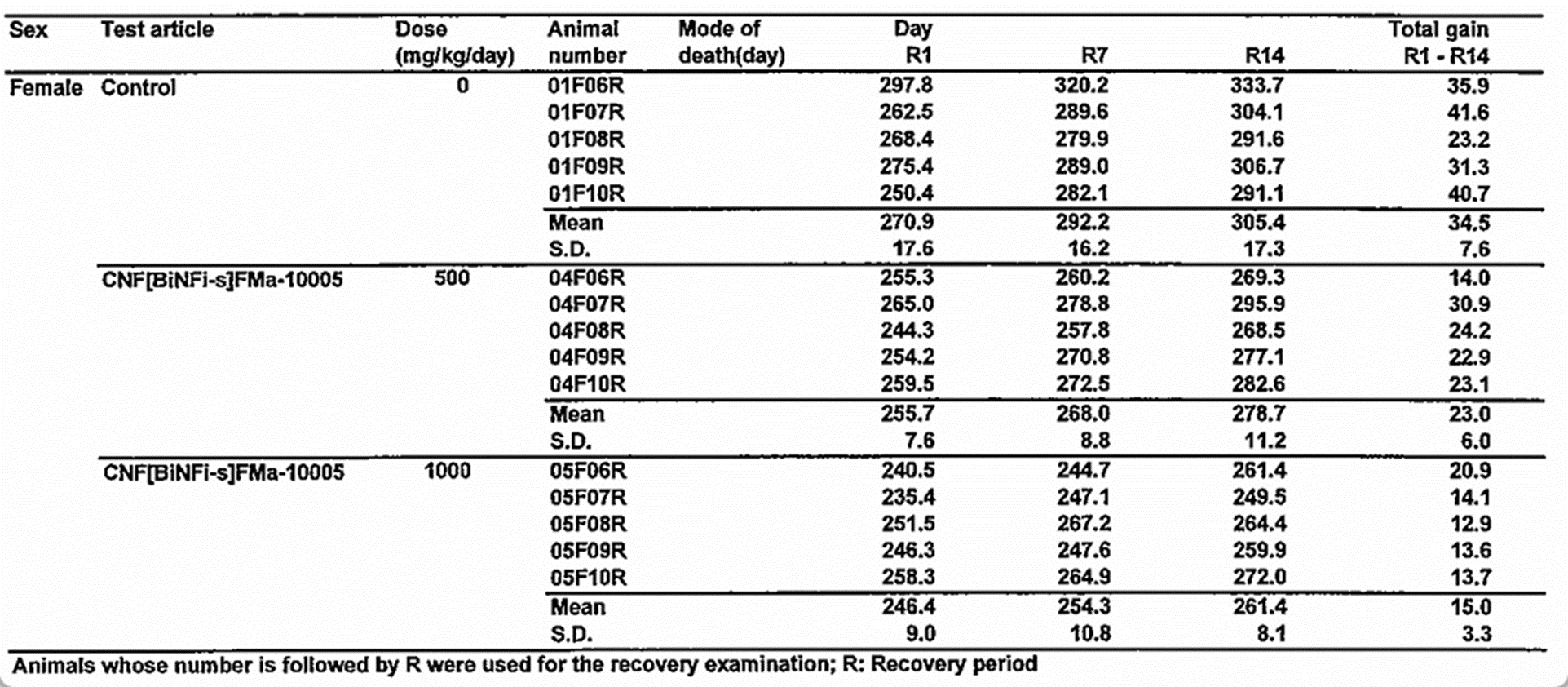


**Table S5.** Urinalysis of male and female rats at week 4.


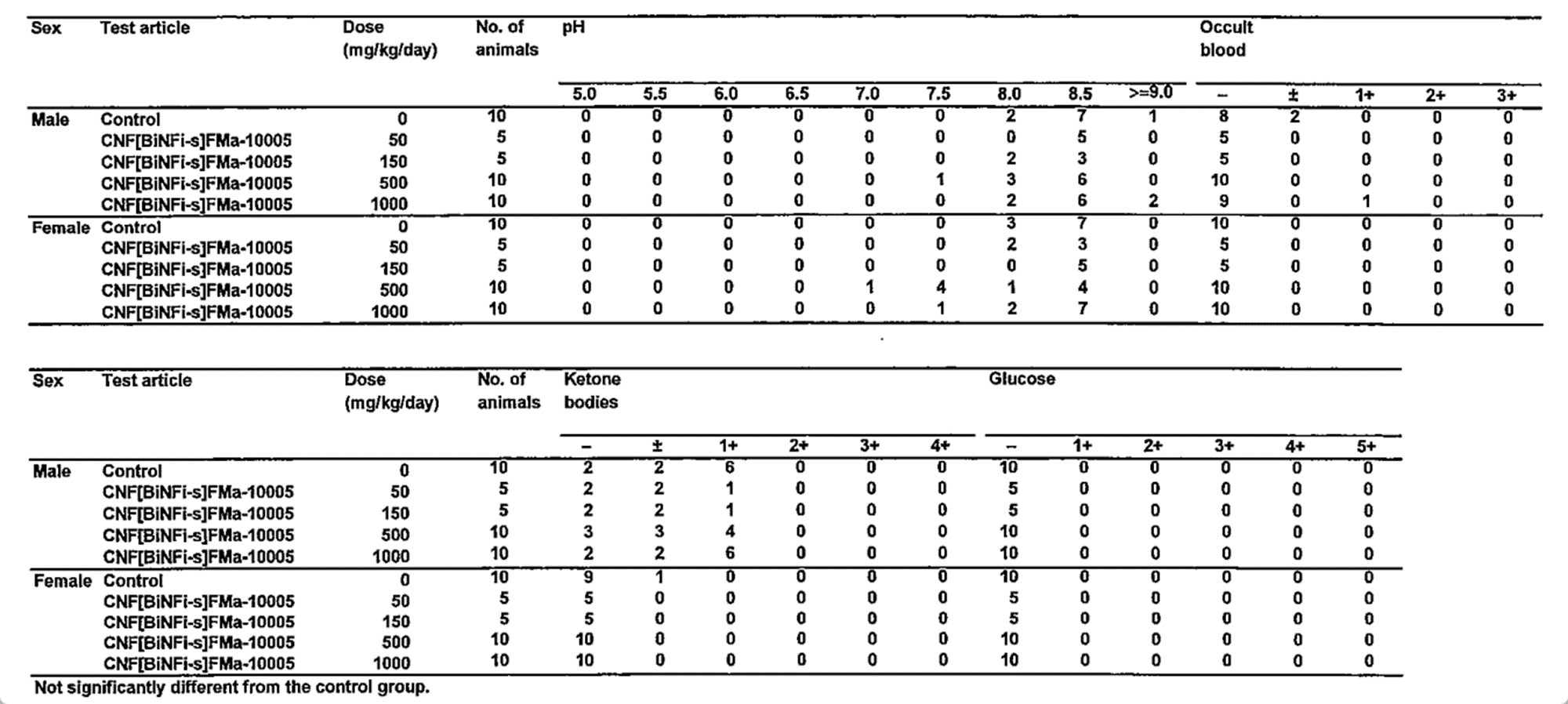


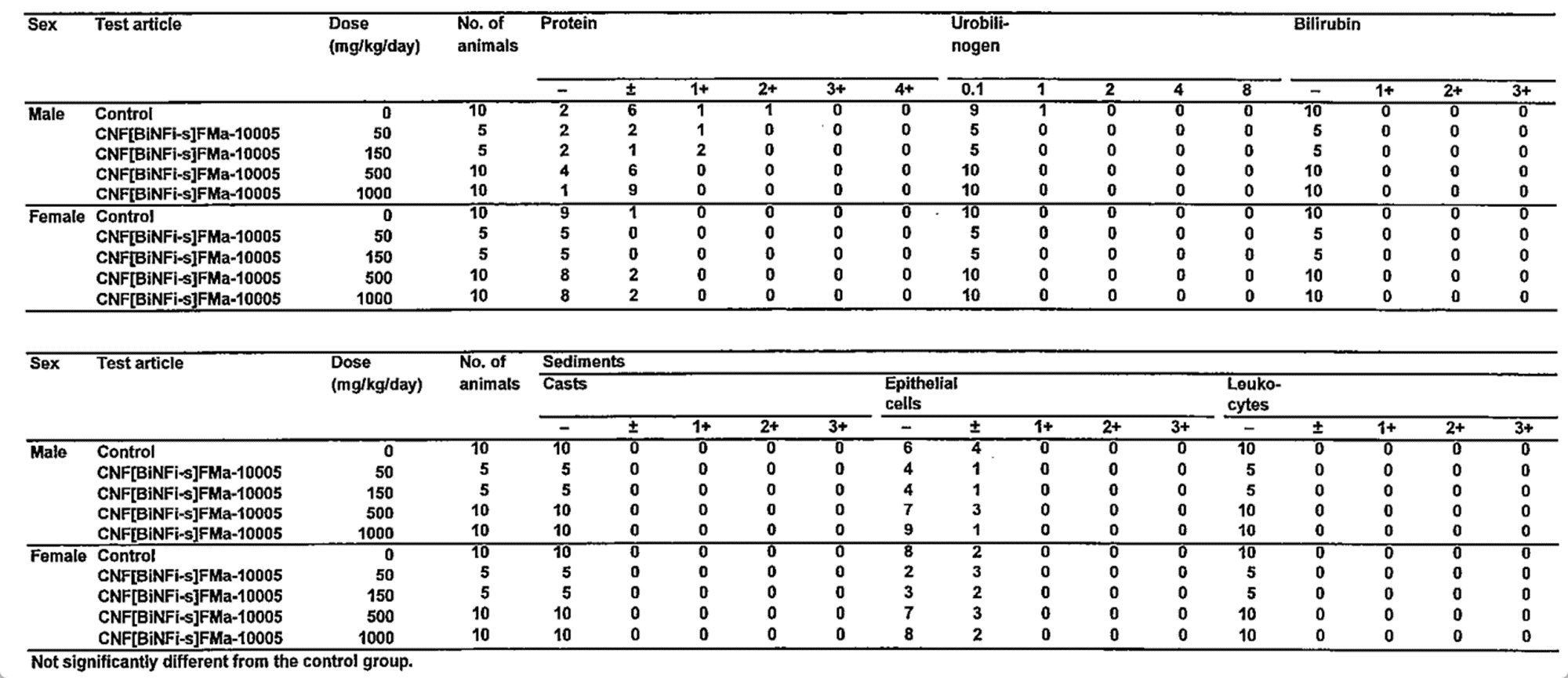


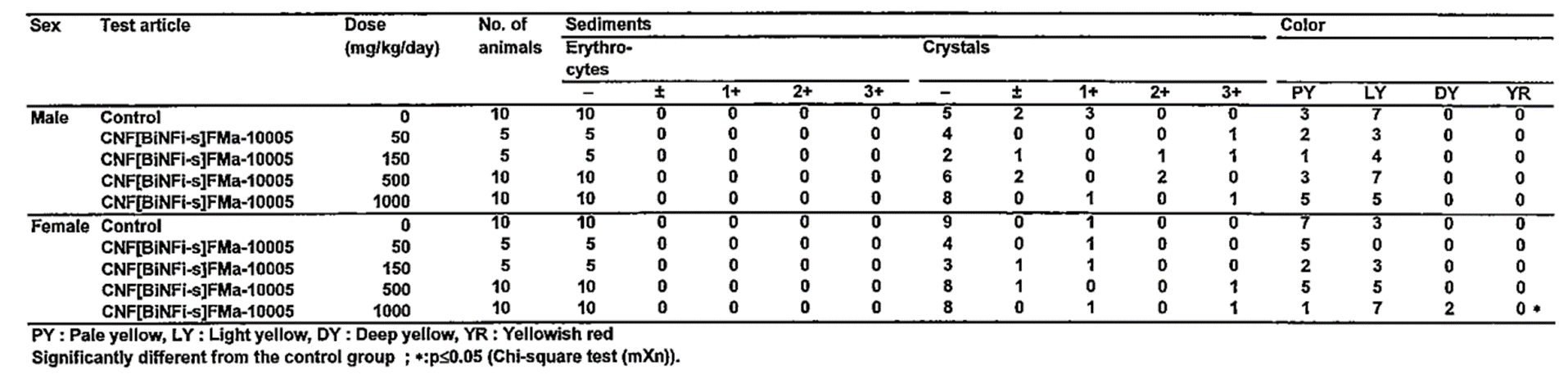


**Table S6.** Urinalysis group mean values (mean ± S.D.) at week 4.


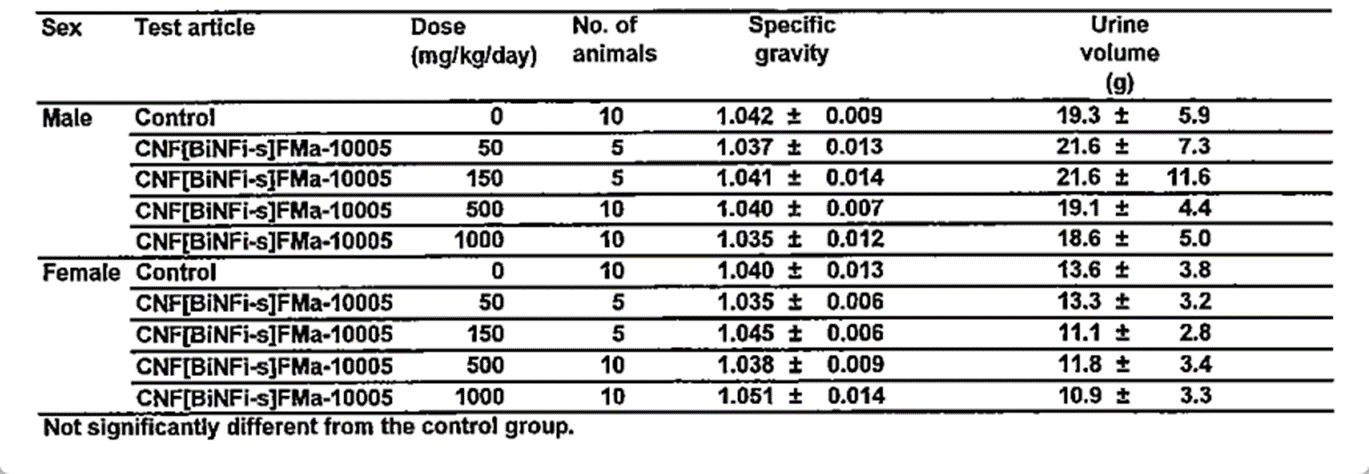


**Table S7.** Urinalysis of male and female rats during the 2-week recovery period.


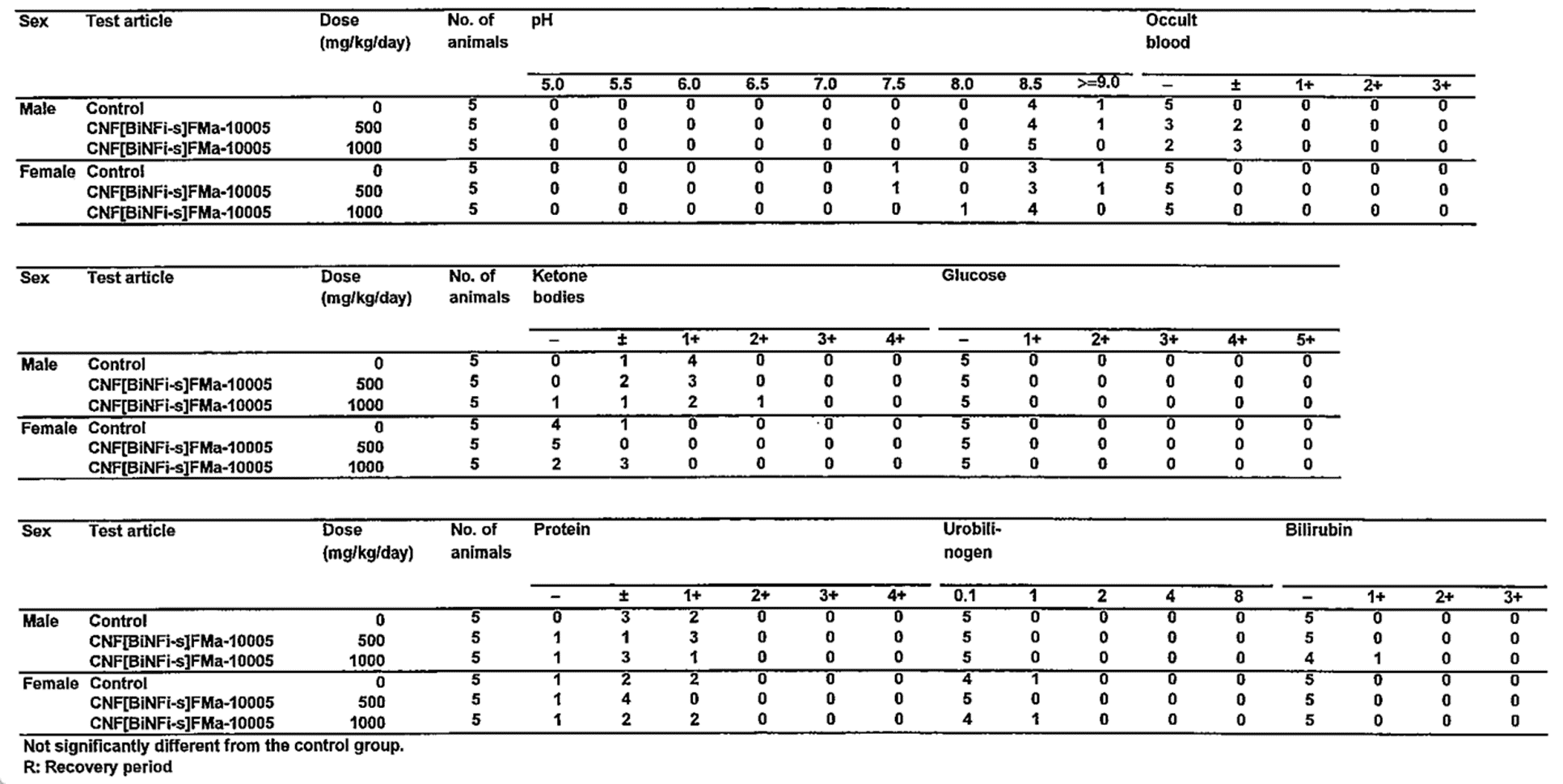


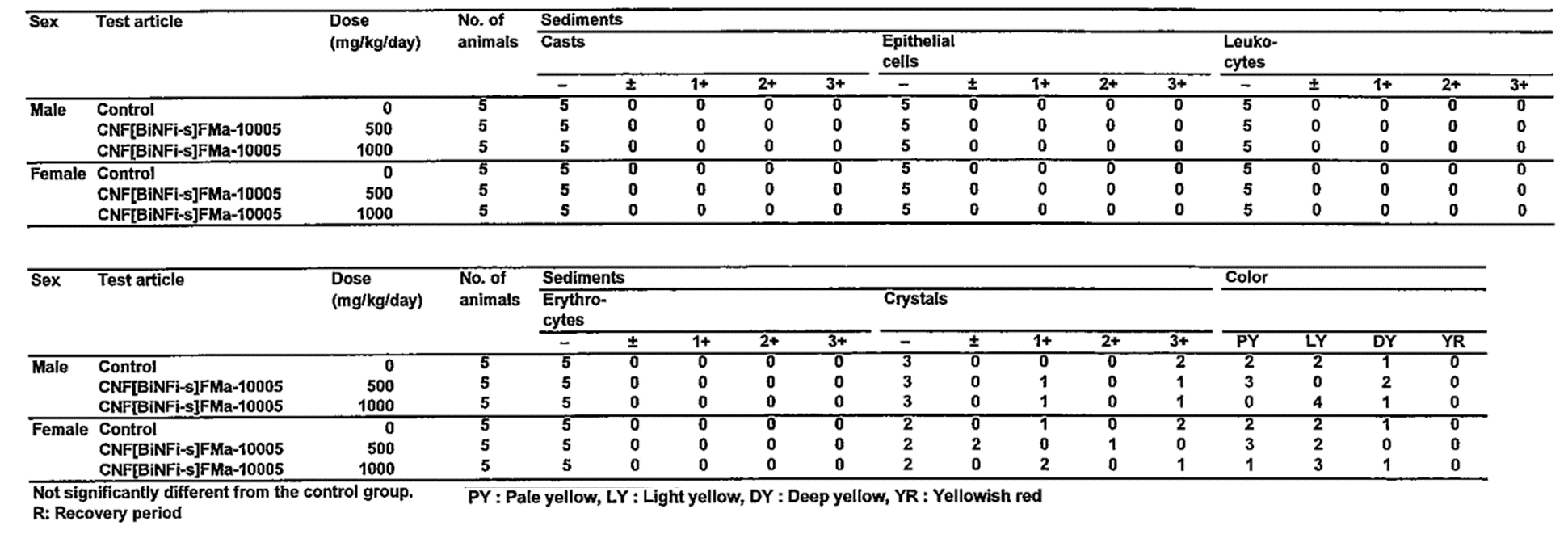


**Table S8.** Urinalysis group mean values (mean ± S.D.) during the 2-week recovery period.


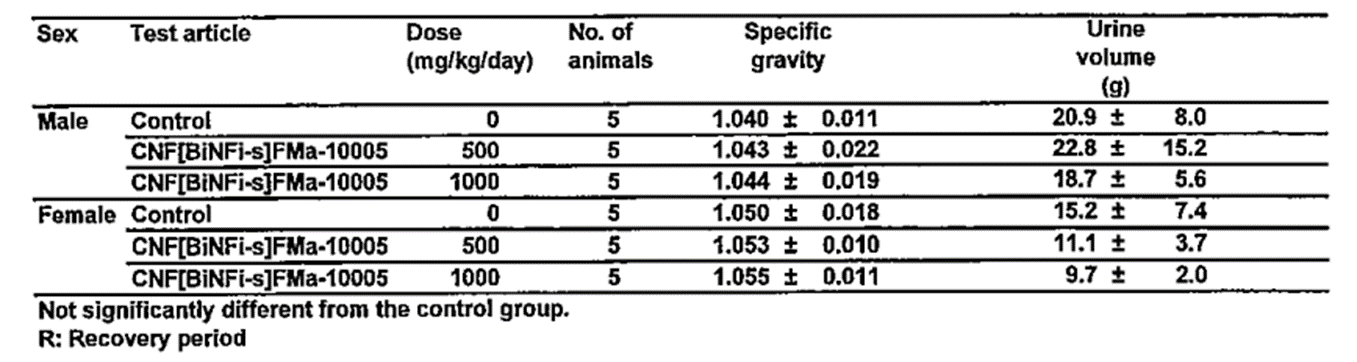


**Table S9.** Hematology group mean values (means ± S.D.) of male and female rats at week 4.


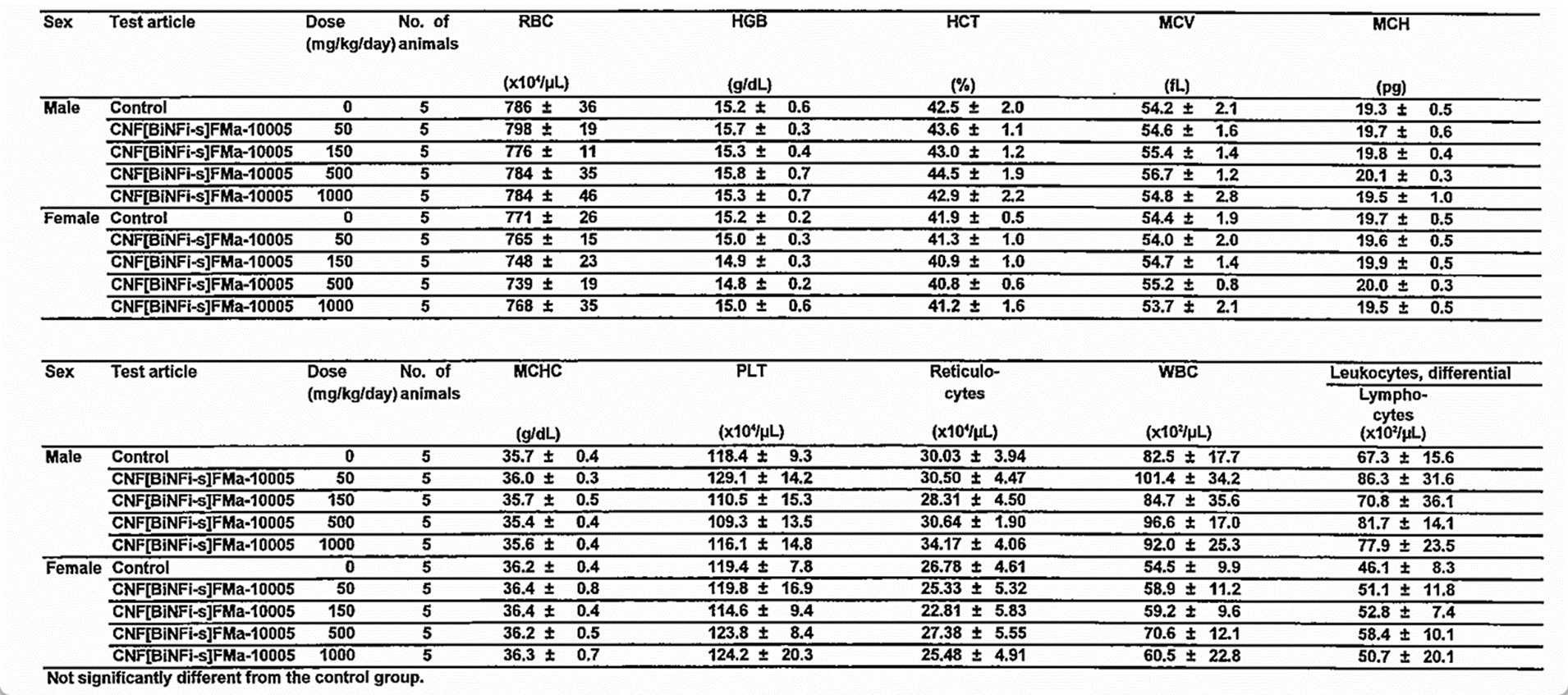


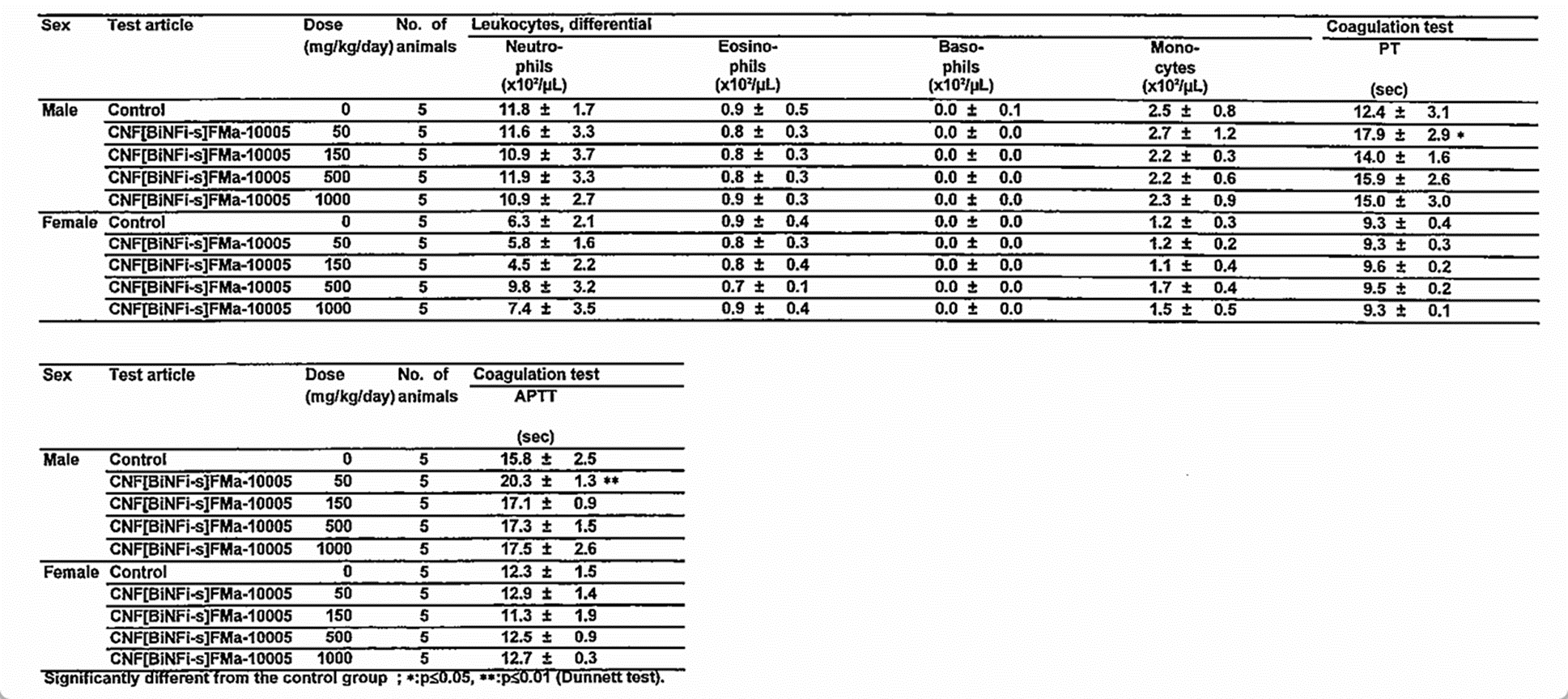


**Table S10.** Hematology group mean values (means ± S.D.) of male and female rats during the 2-week recovery period.


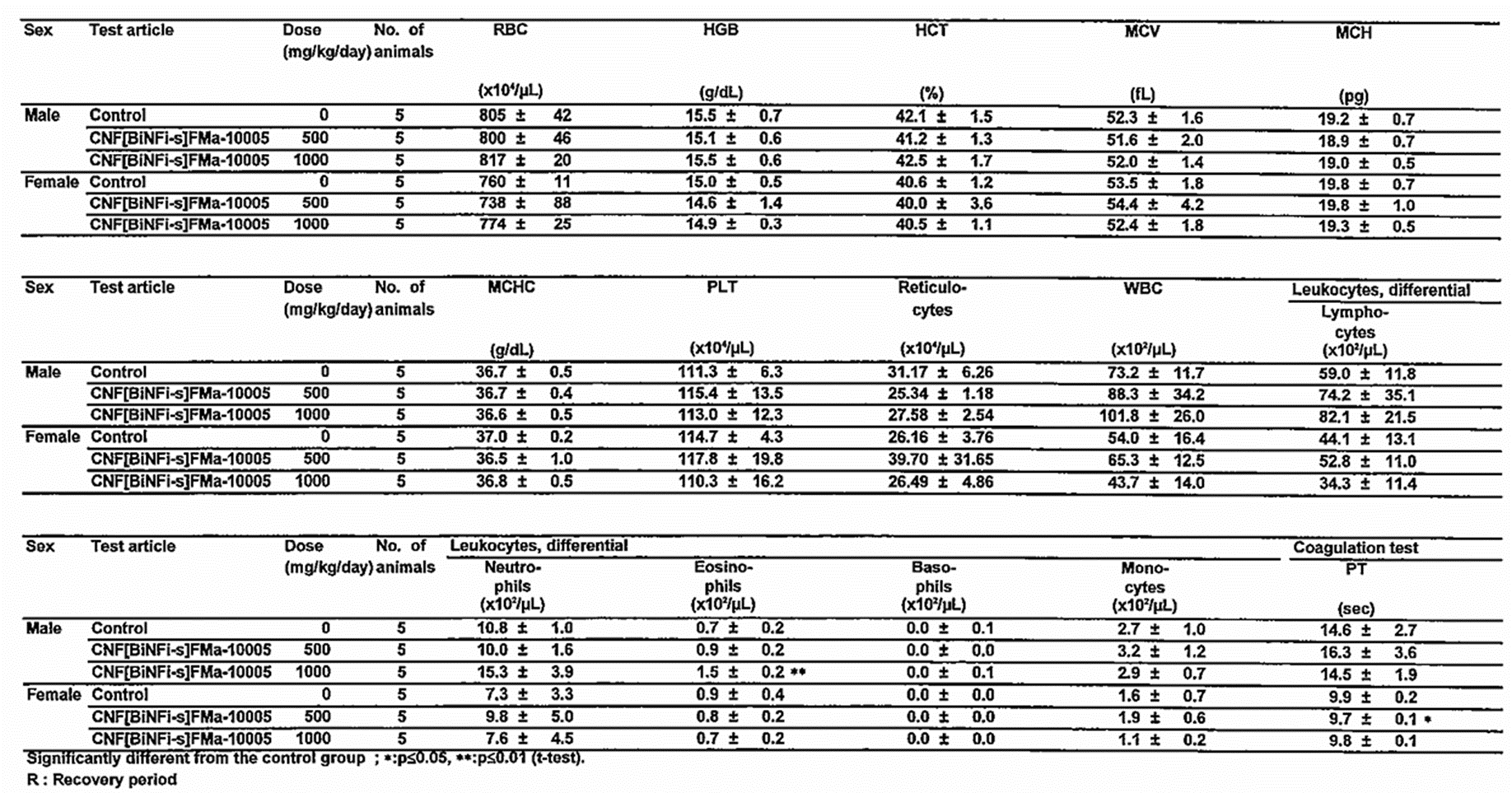


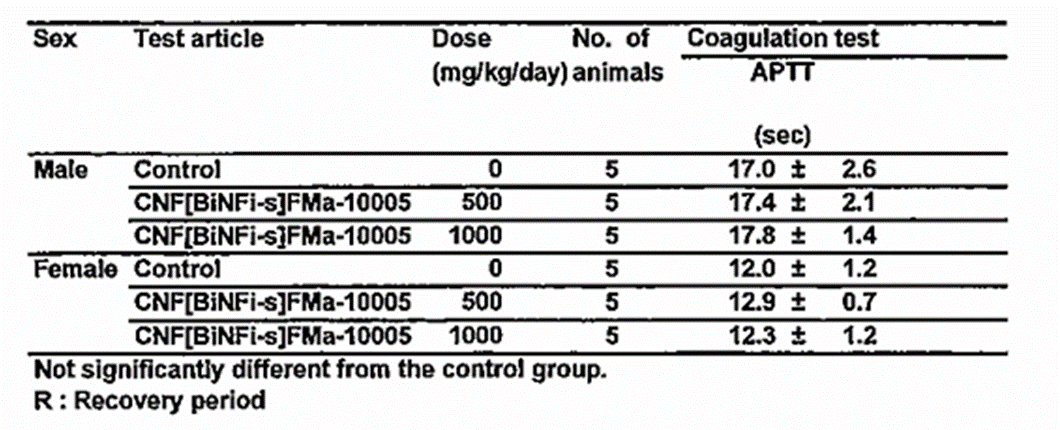


**Table S11.** Clinical biochemistry group mean values (means ± S.D.) of male and female rats at week 4.


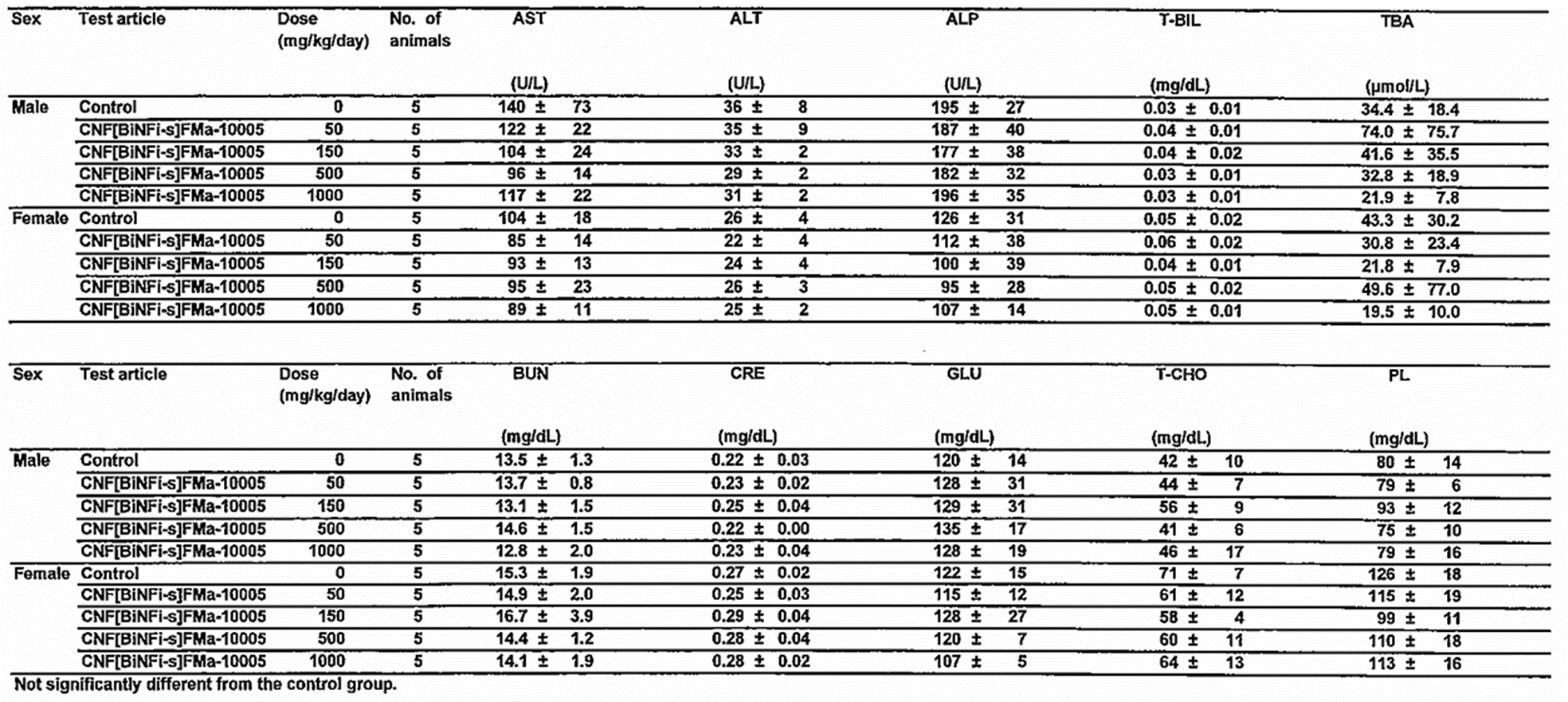


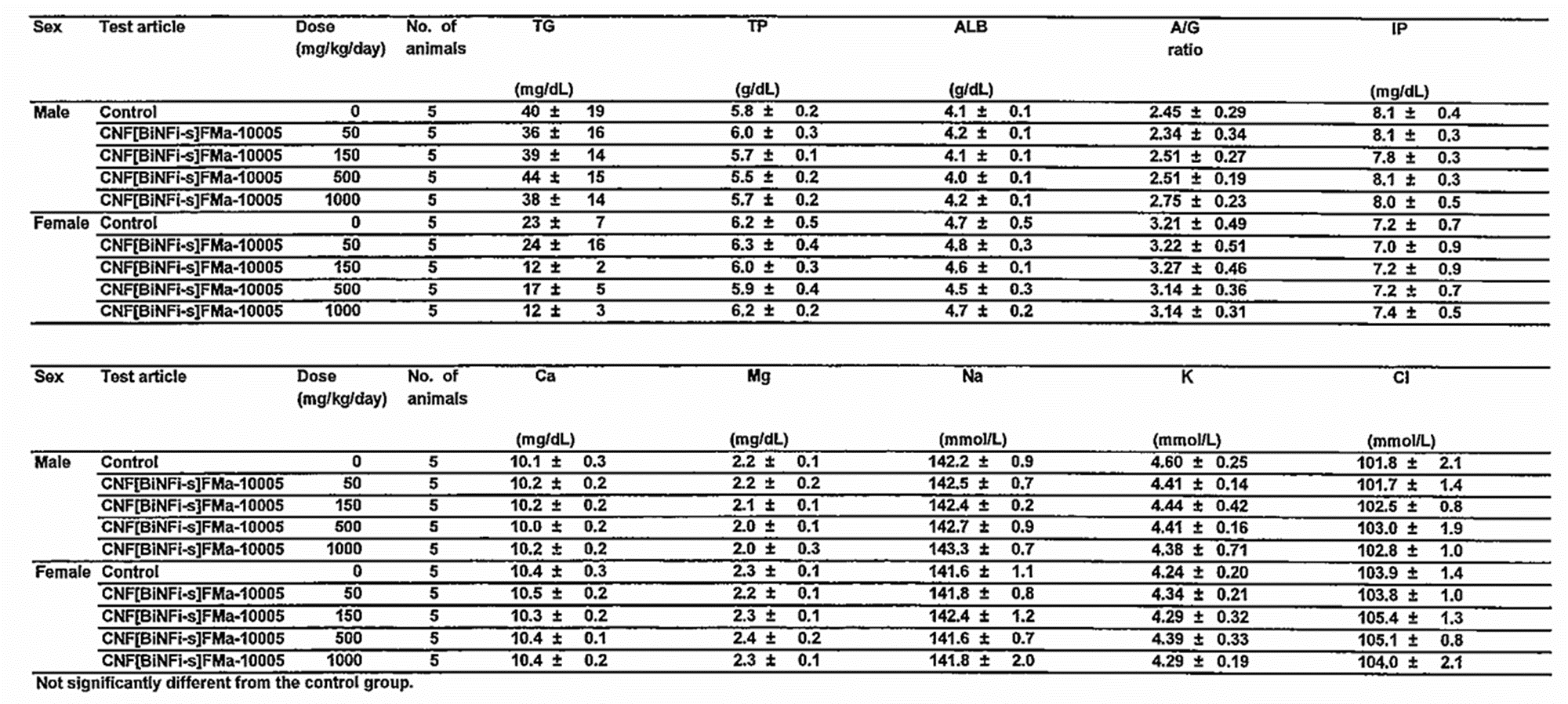


**Table S12.** Clinical biochemistry group mean values (means ± S.D.) of male and female rats during the 2-week recovery period.


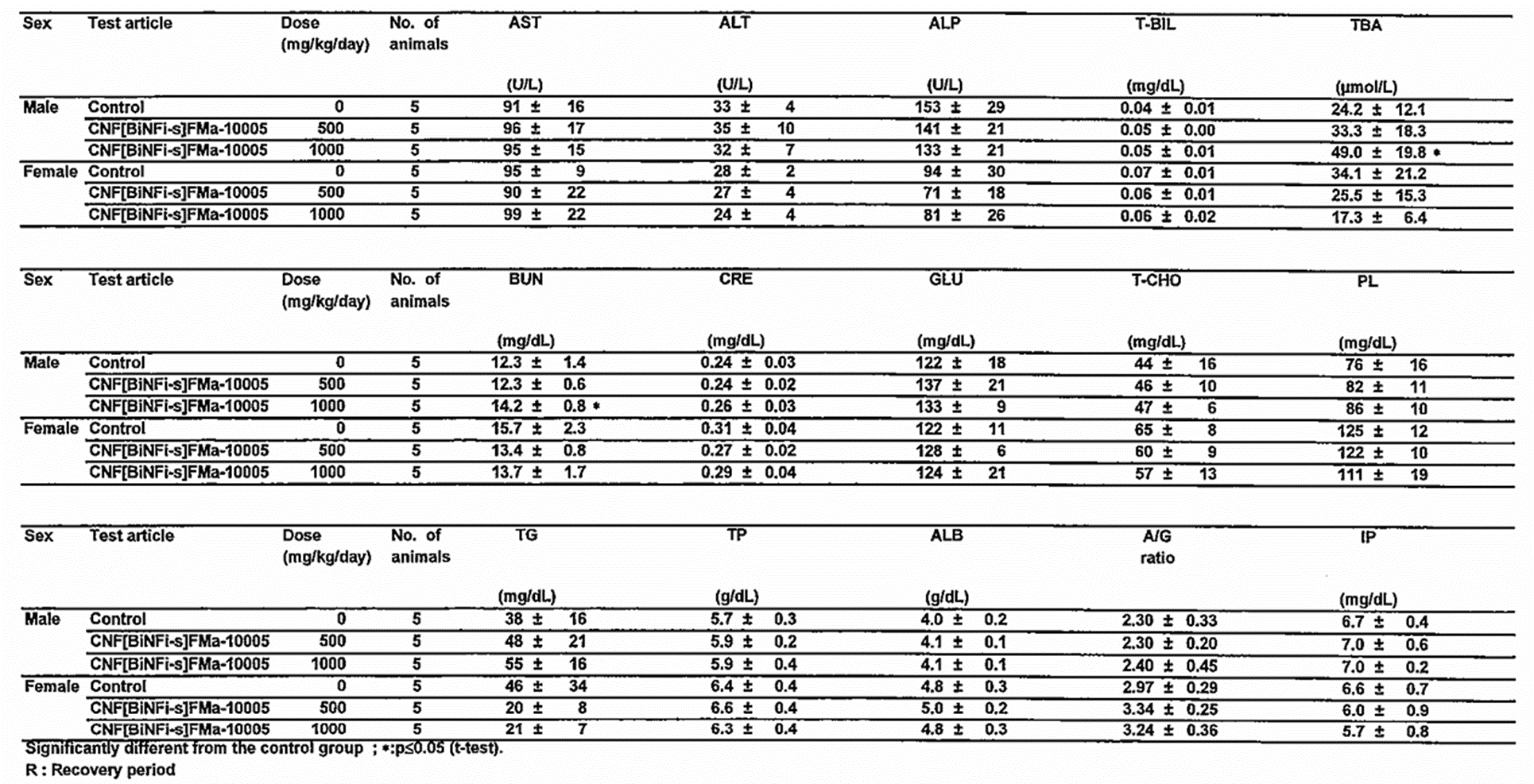


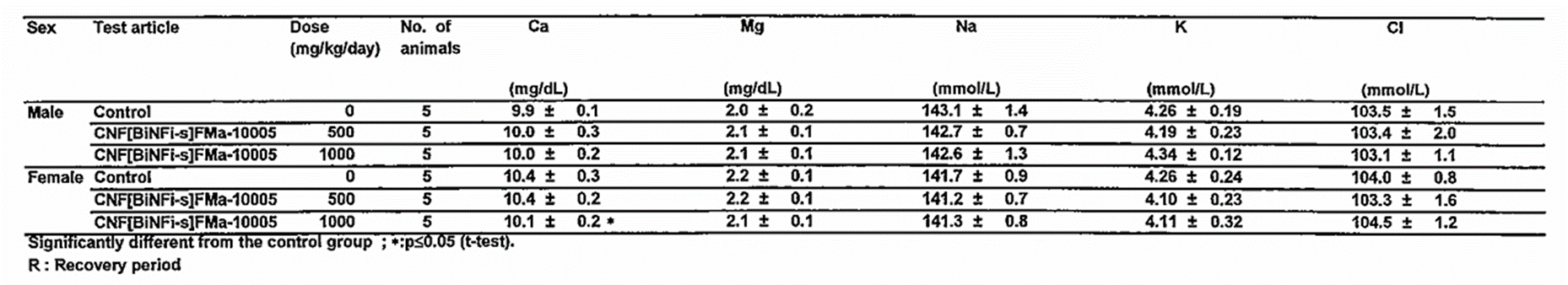


**Table S13.** Points noted in necropsy findings of male rats at week 4.


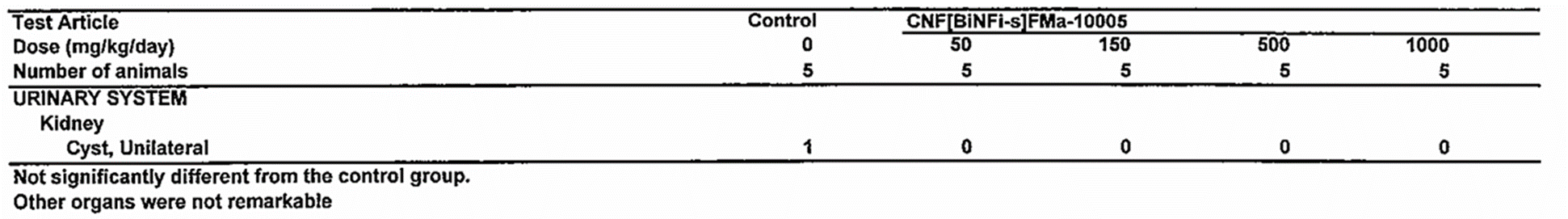


**Table S14.** Points noted in necropsy findings of female rats at week 4.


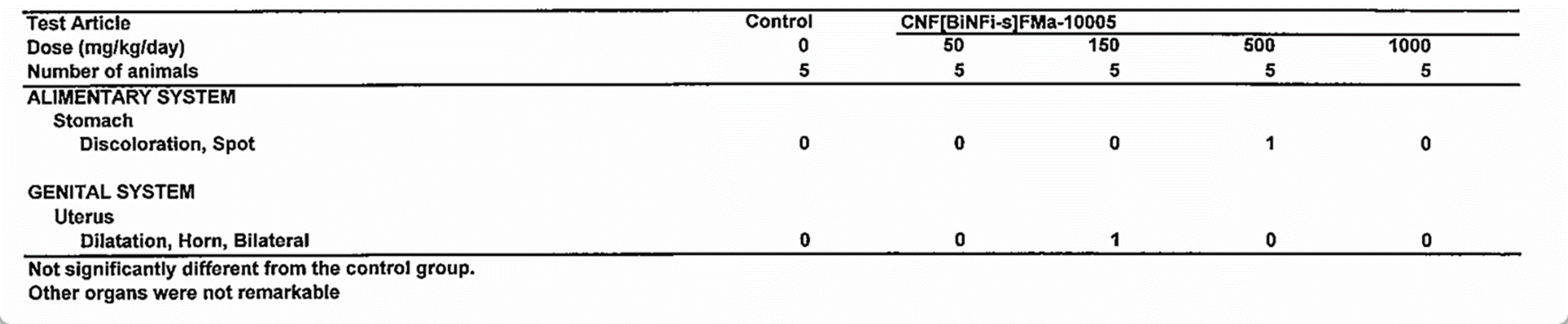


**Table S15.** Points noted in necropsy findings of male rats during the 2-week recovery period.


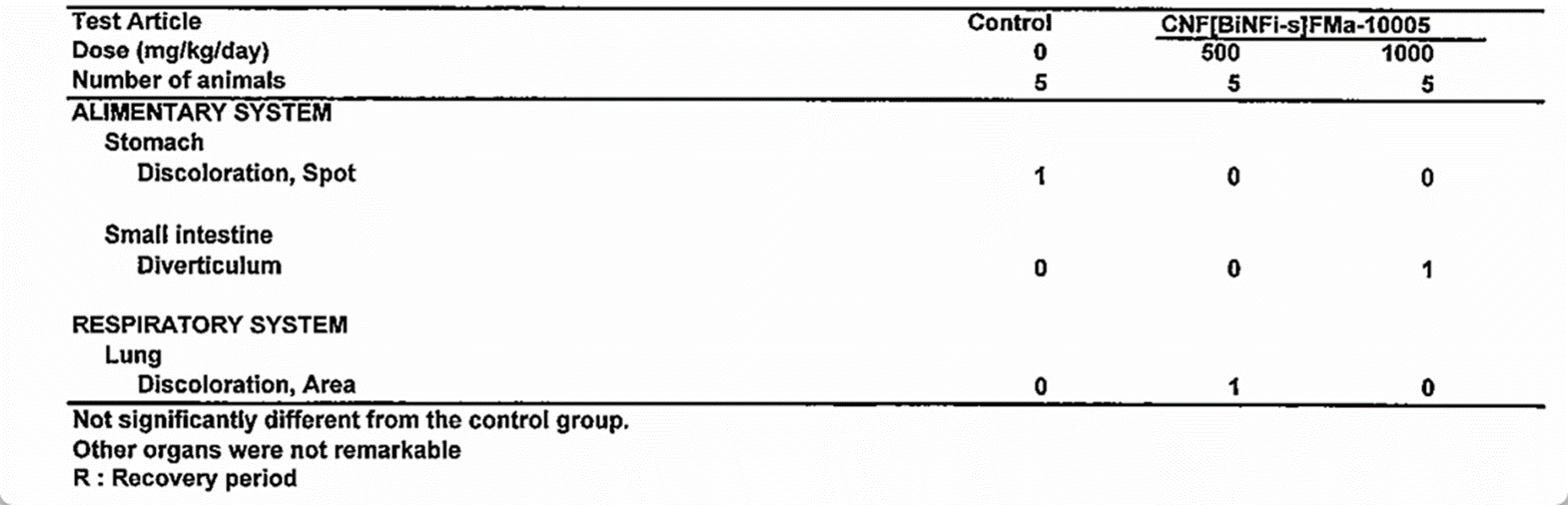


**Table S16.** Points noted in necropsy findings of female rats during the 2-week recovery period.


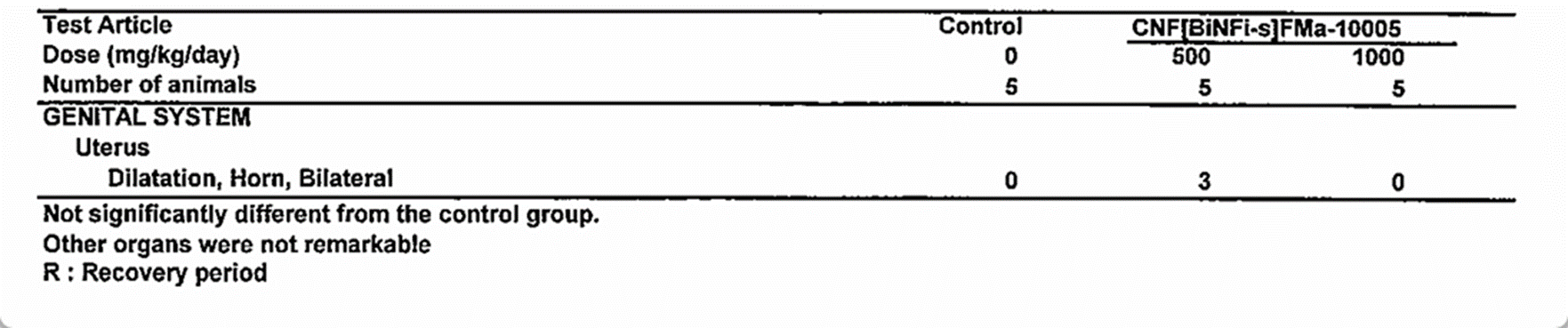


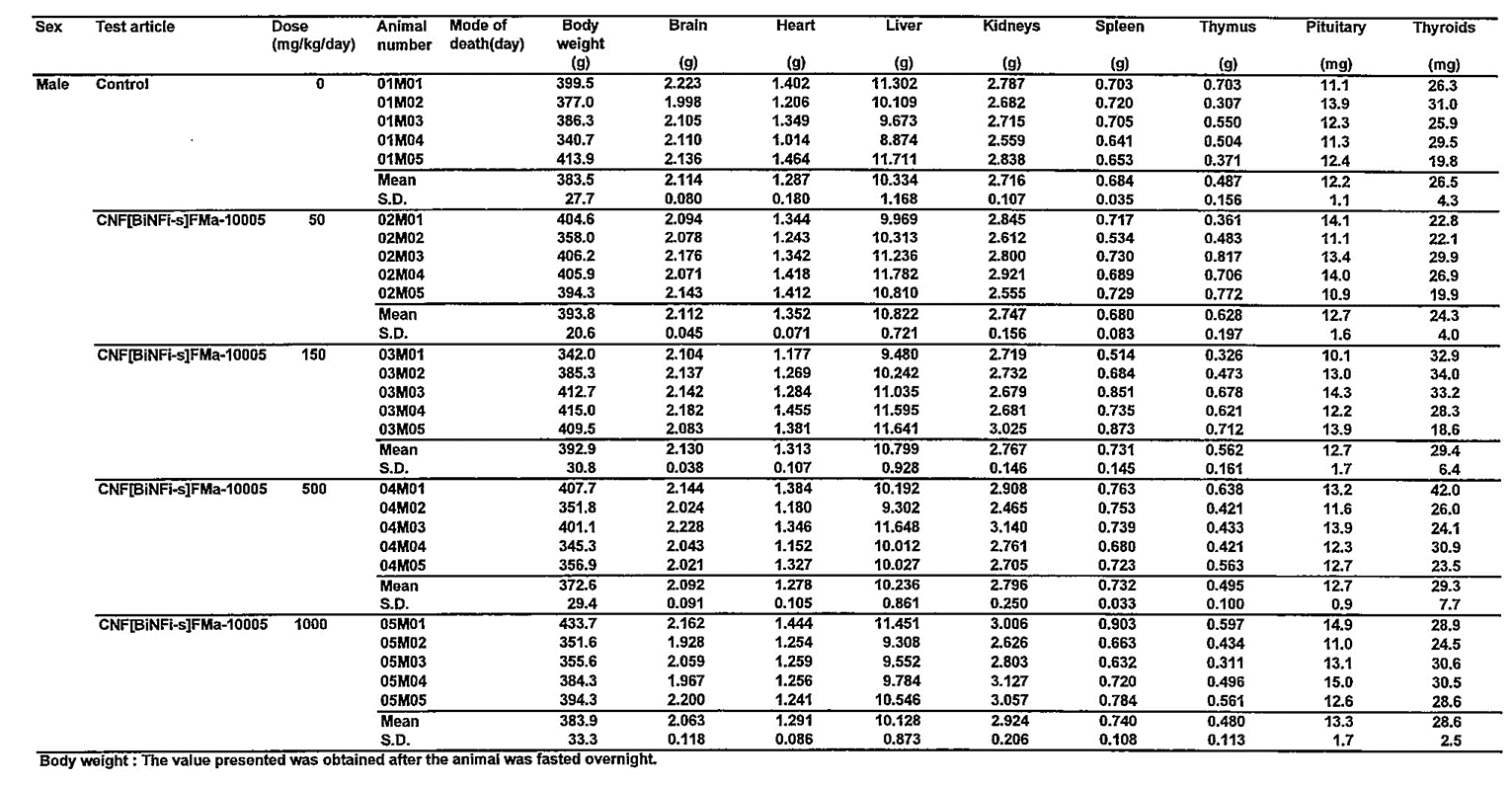
**Table S17.** Individual organ weights of male rats at week 4.

**
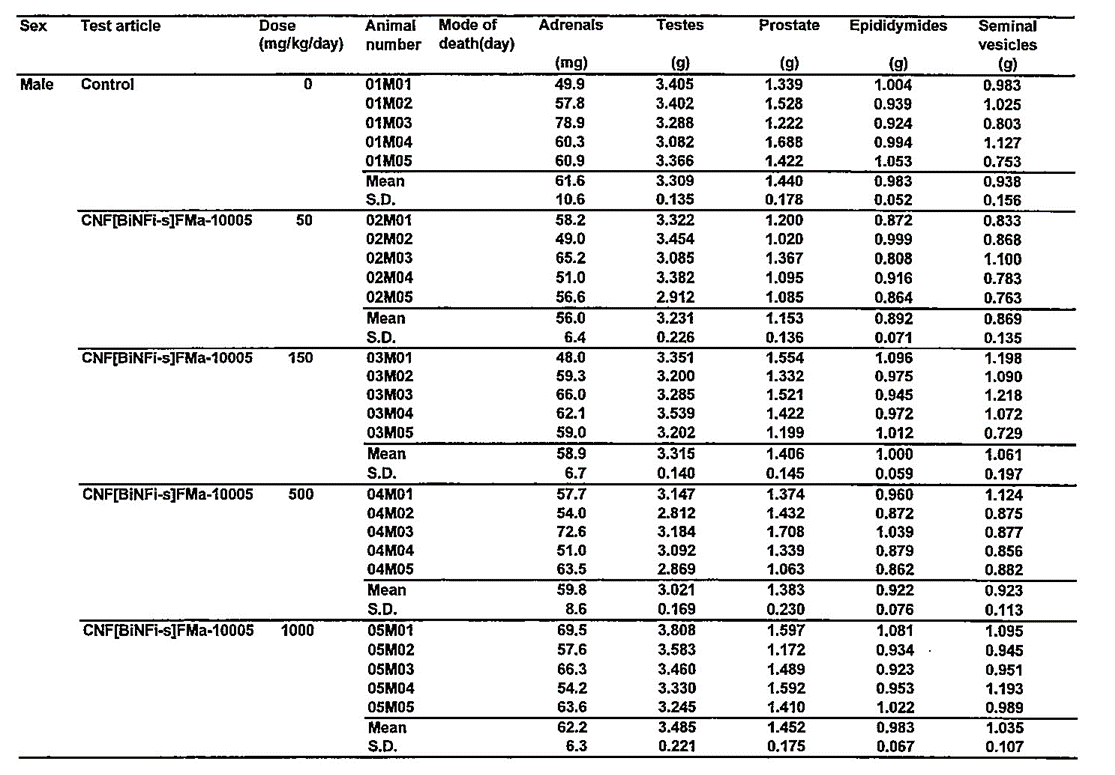
**

**Table S18.** Individual organ weights of male rats during the 2-week recovery period.


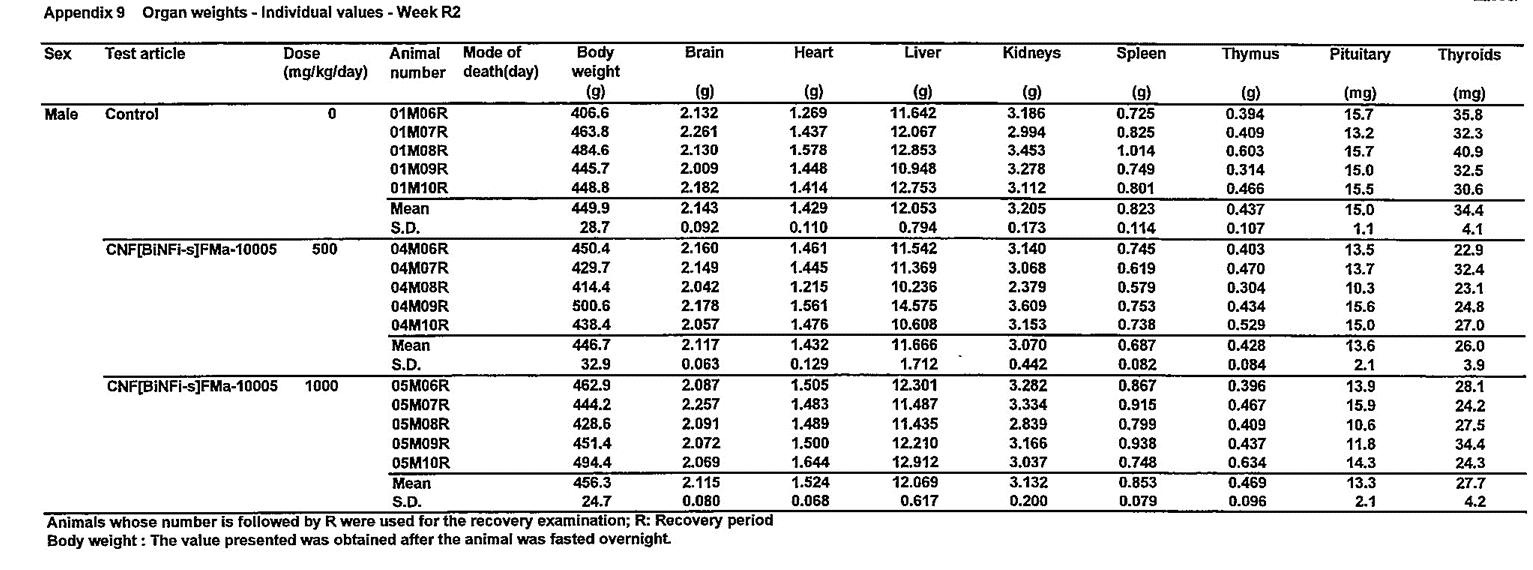


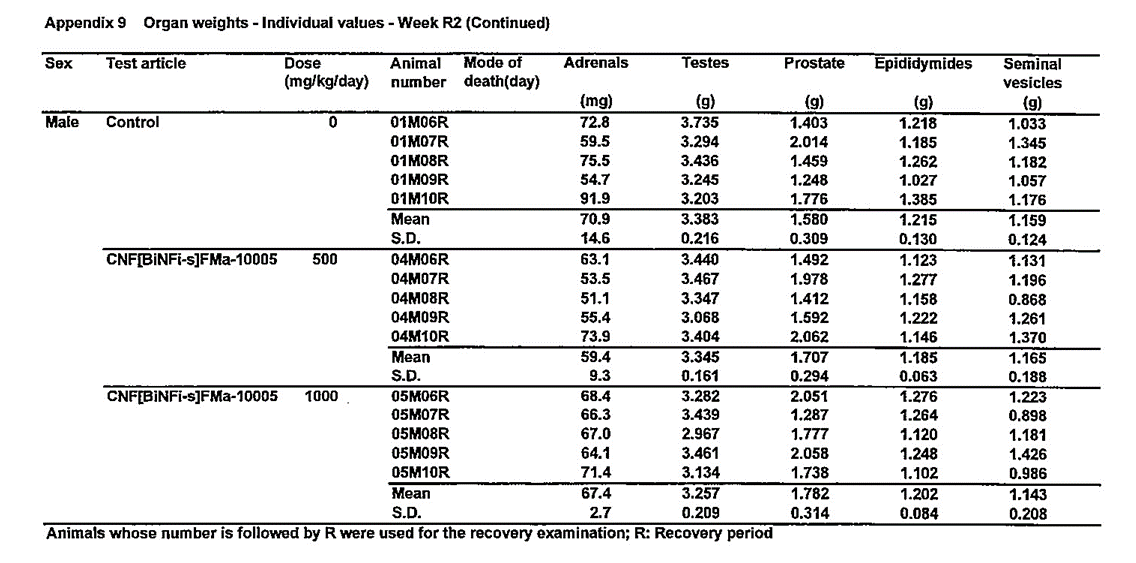


**Table S19.** Individual organ weights of female rats at week 4.


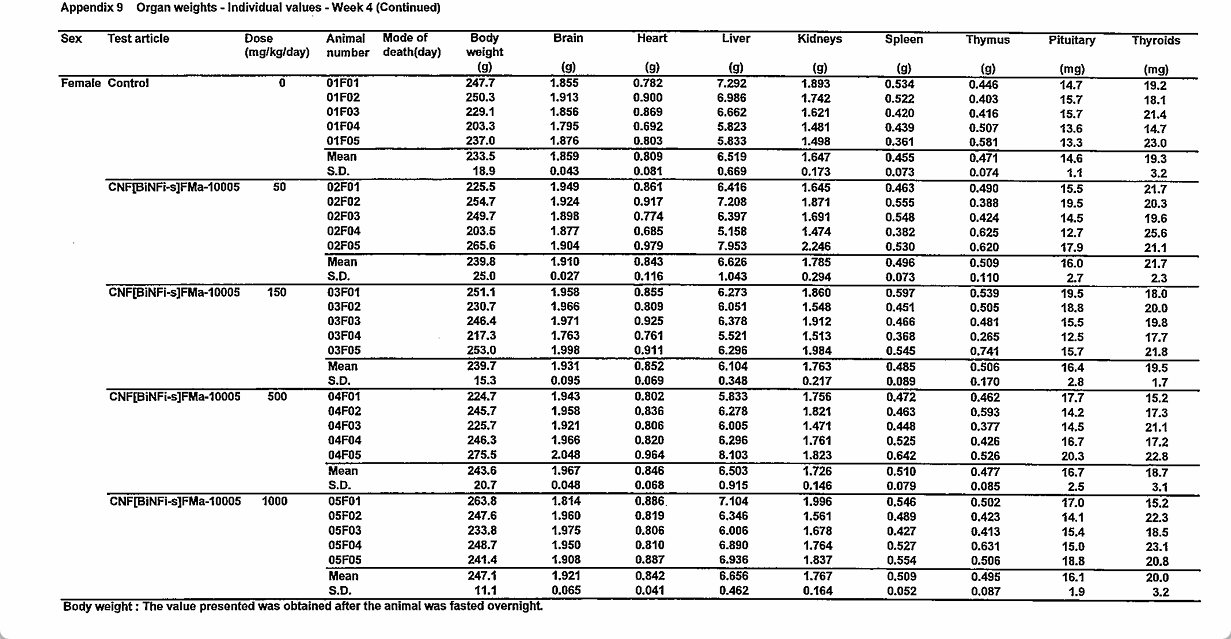


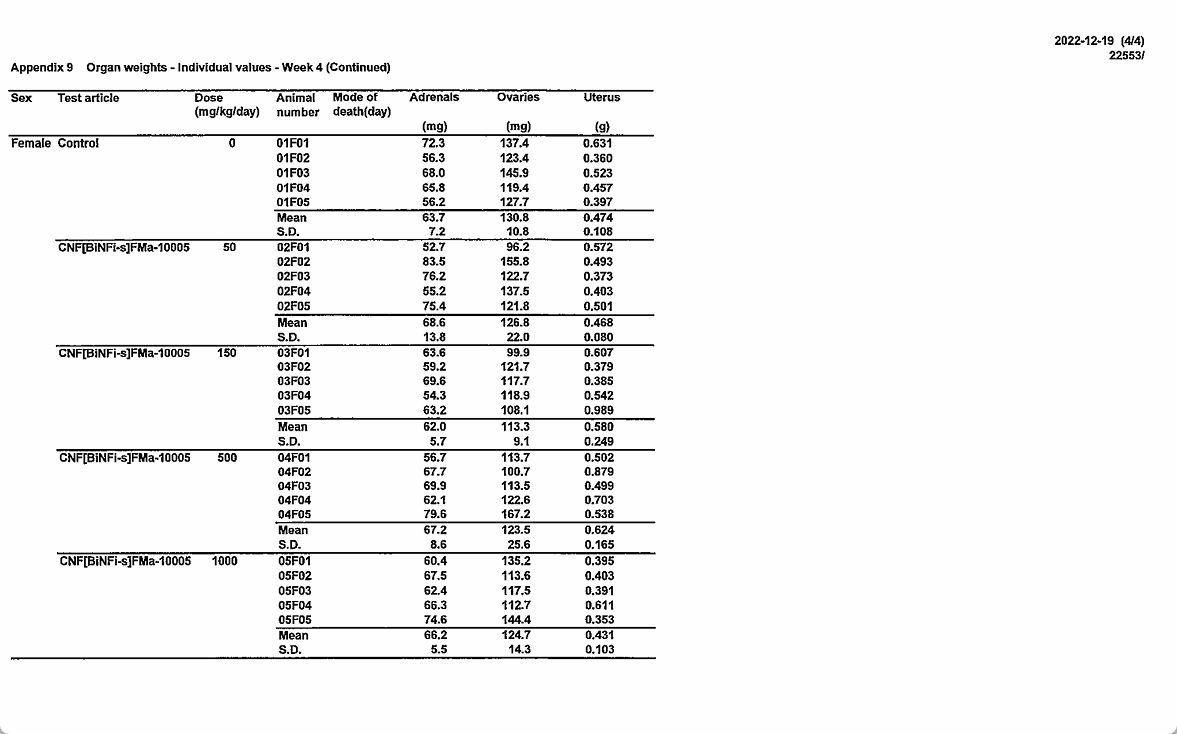


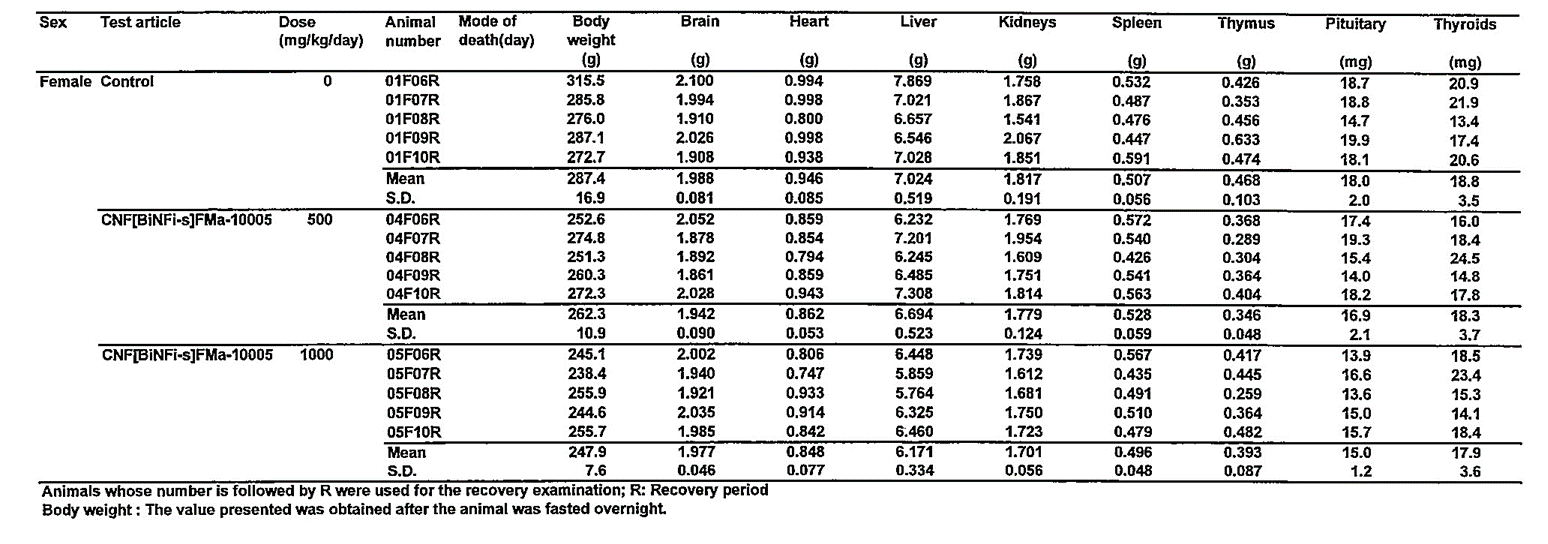
**Table S20.** Individual organ weights of female rats during the 2-week recovery period.

**
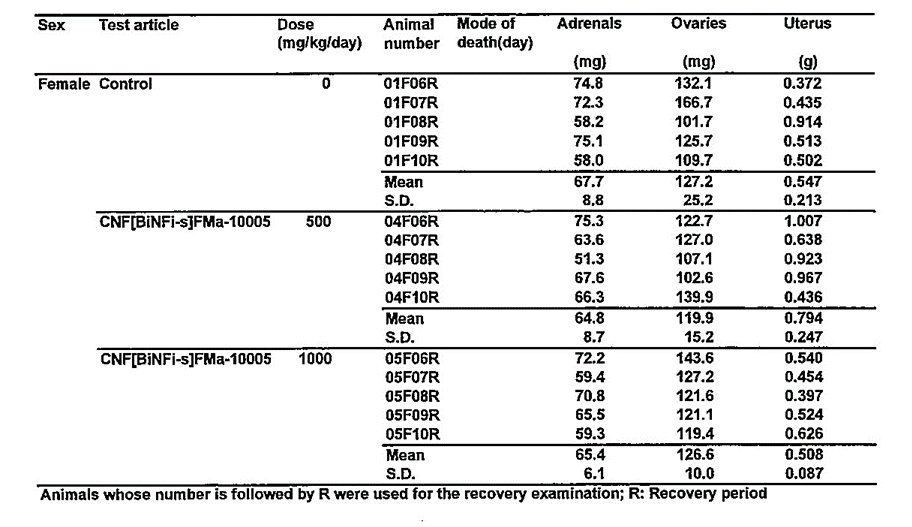
**

**Table S21.** Histopathological findings of male rats at week 4.


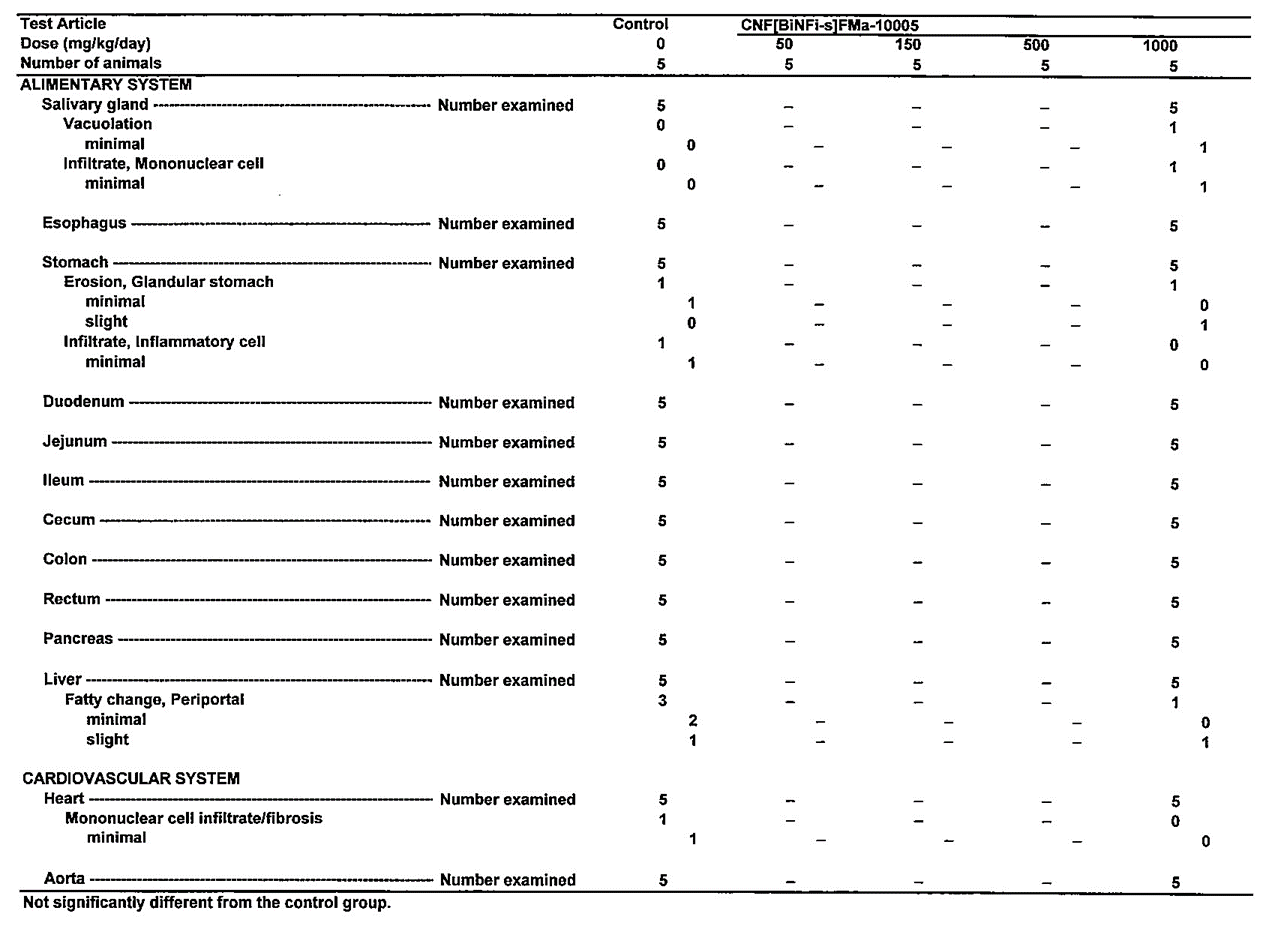


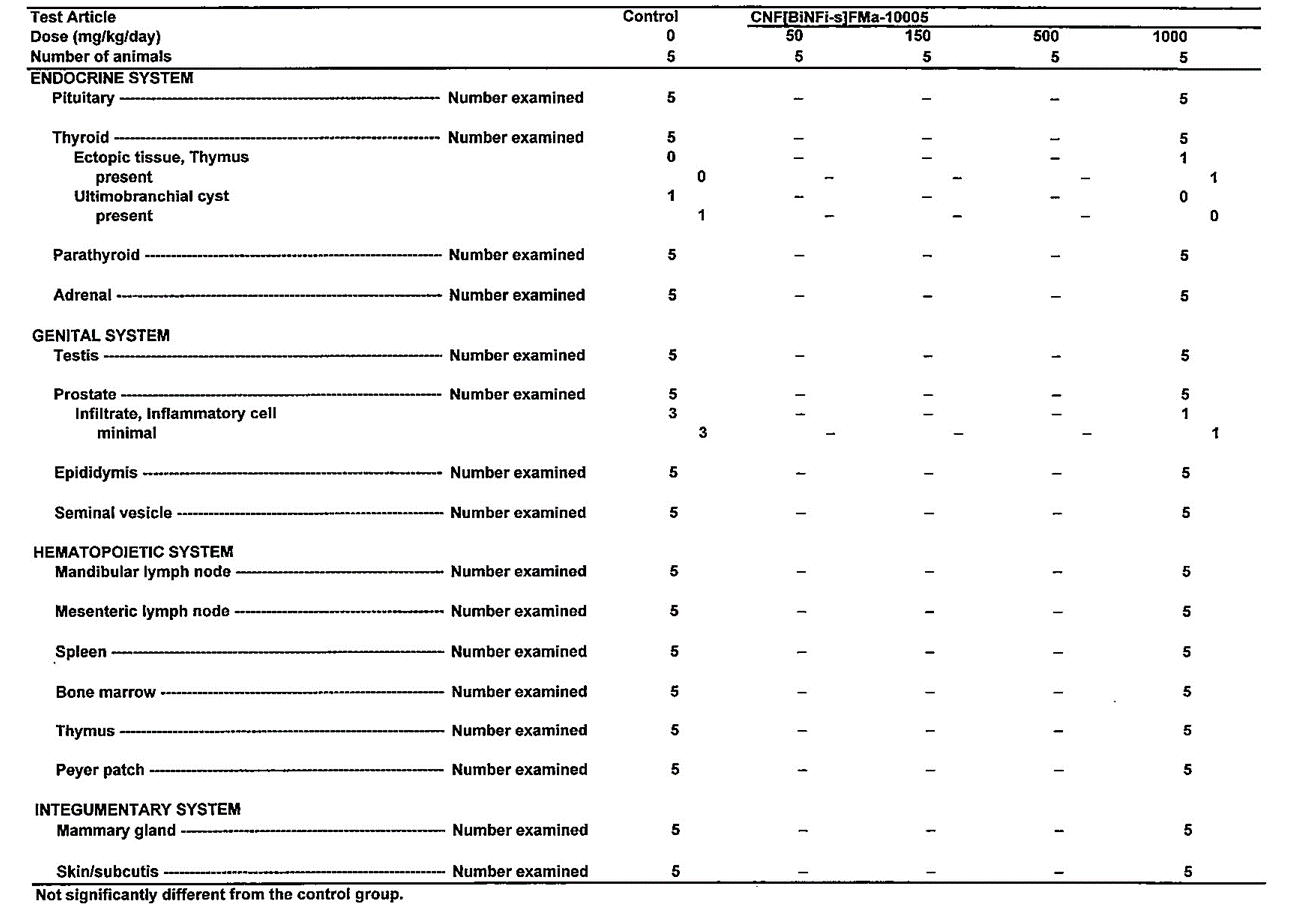


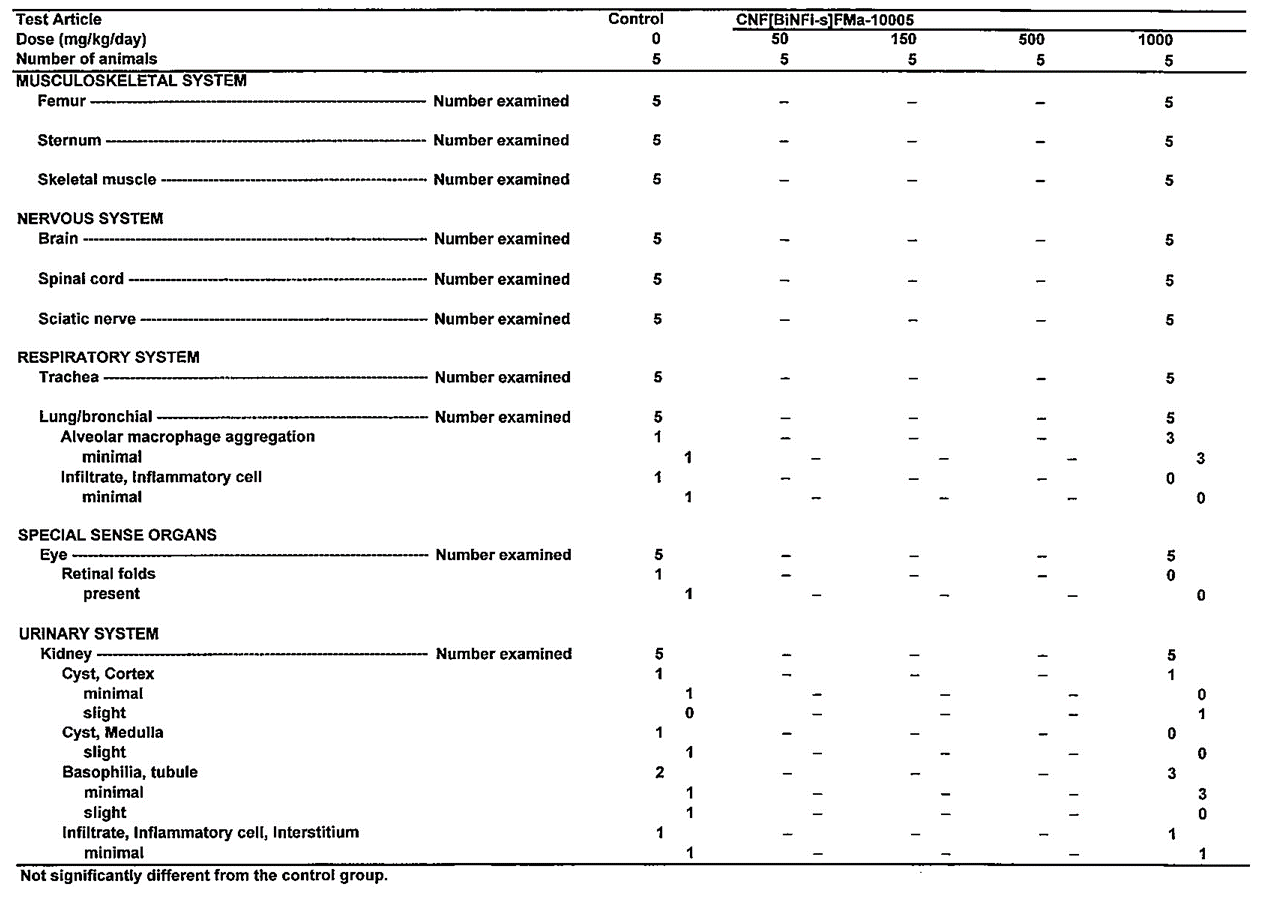


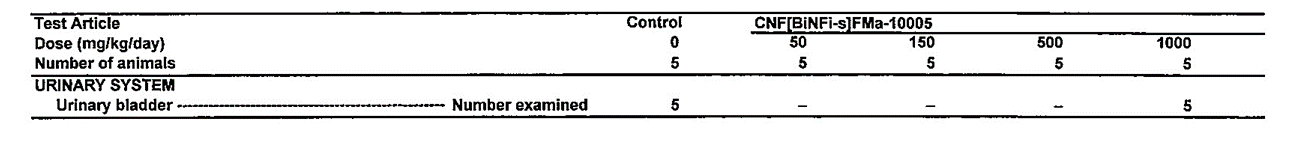


**Table S22.** Histopathological findings of female rats at week 4.


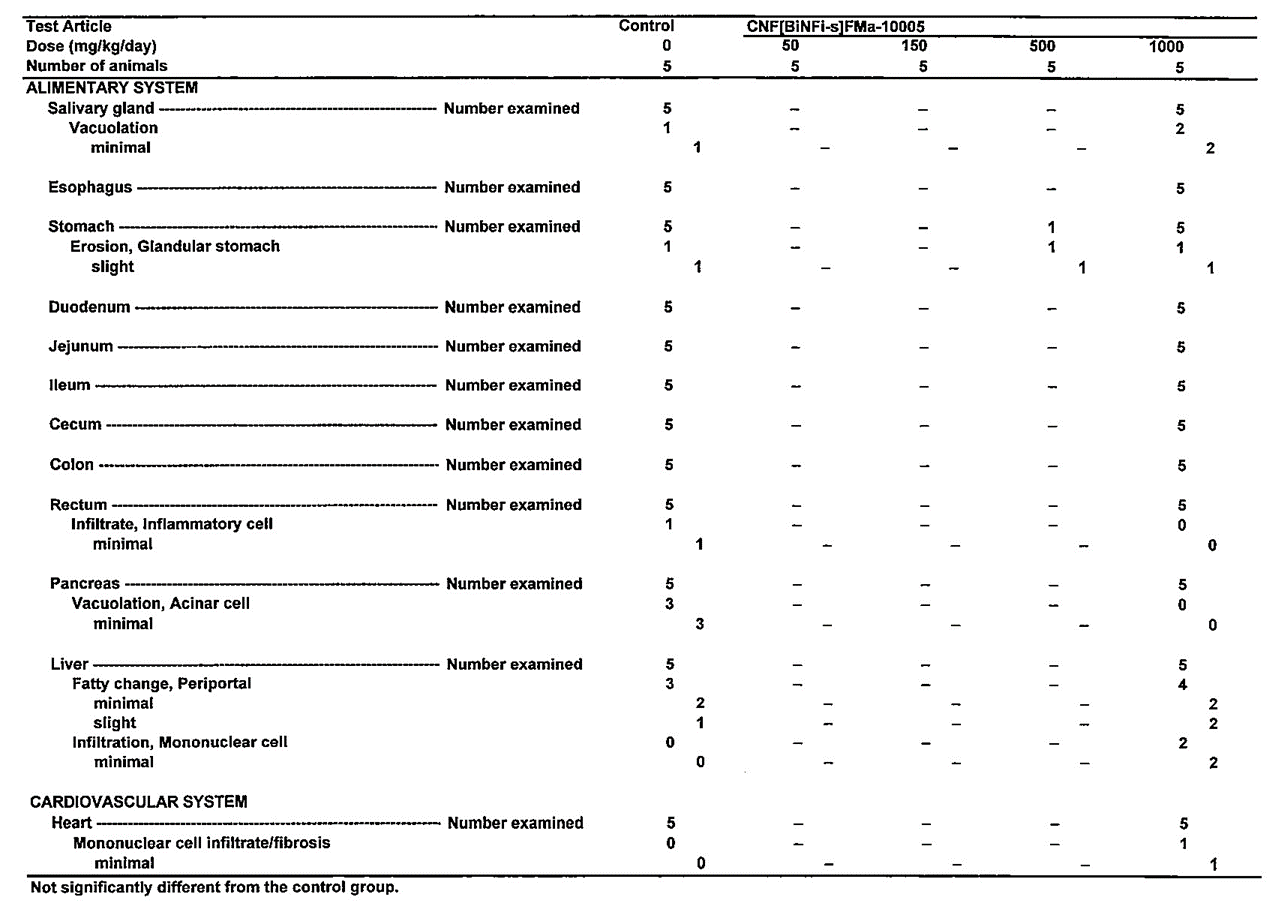


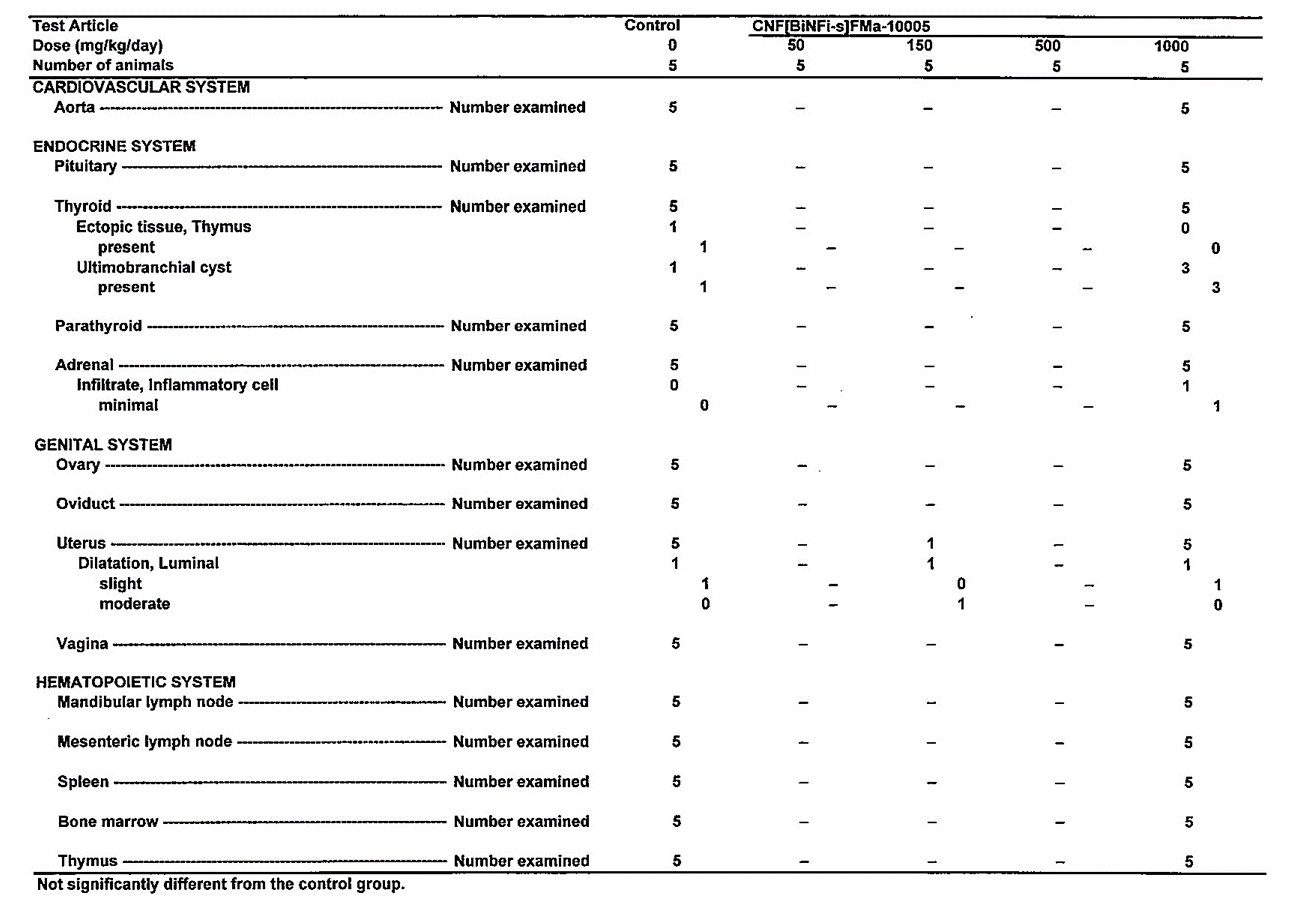


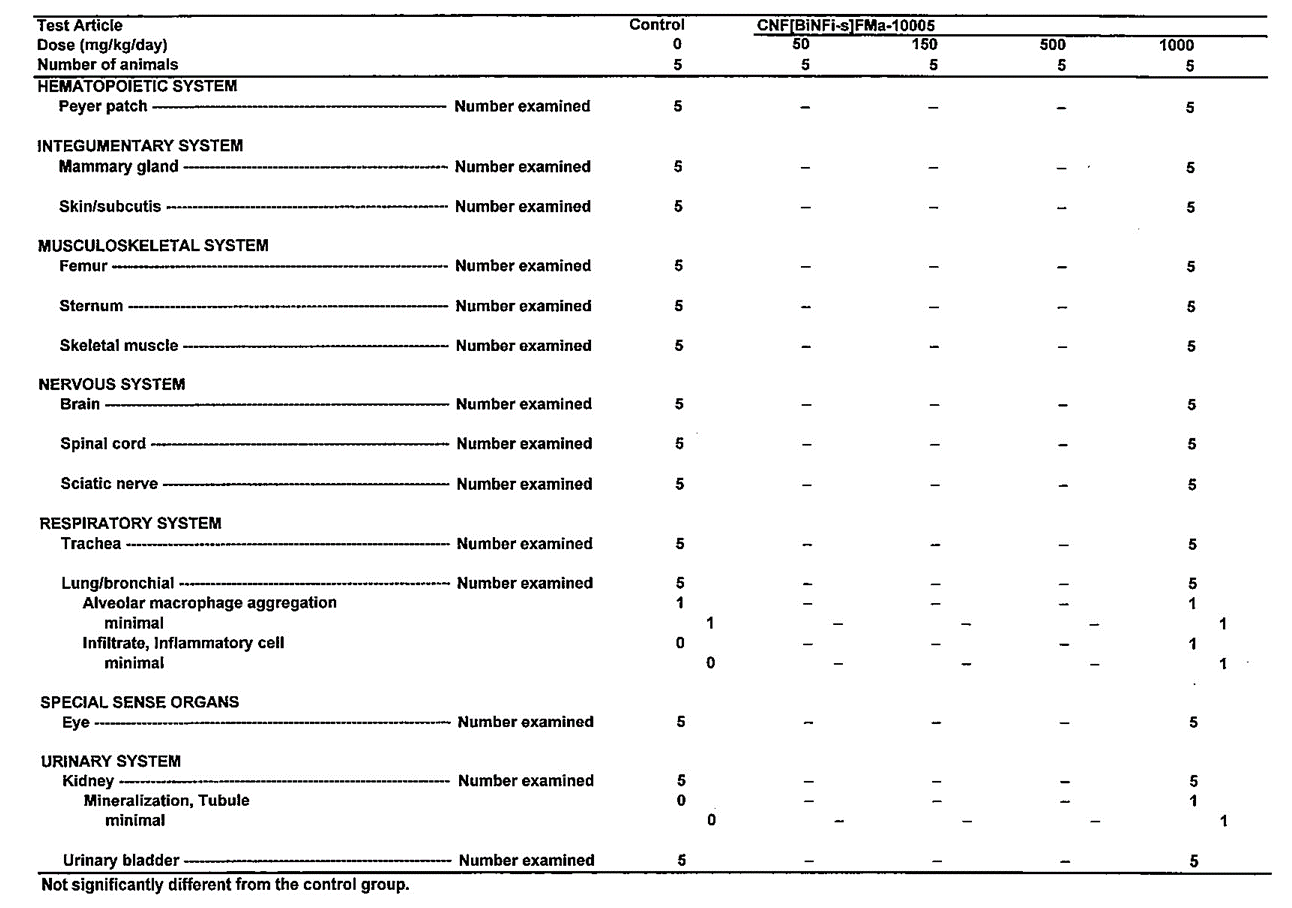

Supplement: Supplementary file 1 [file nanomaterials-14-01082-s001.zip › nanomaterials-3037437-supplementary.docx]
